# Beef Tea and Meat Preparations

**Published:** 1902-01-25

**Authors:** Marcus R. P. Dorman


### THE INSTITUTIONAL WORKSHOP.

### BEEF TEA AND MEAT PREPARATIONS.

By MARCUS R. P. DORMAN, M.D.

I.—INTRODUCTORY.

IN the present series of articles it is proposed to discuss the relative advantages of the use of beef tea and commercial meat preparations in the treatment of disease. Beef tea may be defined as an infusion of beef in water, and meat extracts are regarded by Liebig as "beef tea made from fresh meat—not roasted—in the purest state condensed to the consistency of a thick honey, to which nothing whatever is added by the manufacturer." Many of the preparations of meat on the market cannot come under the heading of extracts since they are not simple beef tea and often have other matter added. To avoid confusion therefore in these articles, we shall use the term beef tea to indicate a home-brewed solution, meat extract when we are referring to a manufactured article which in general principle fulfils the terms of Liebig's definition, and meat preparation when we are speaking of the numerous meat juices, soups, bouillons and other forms of prepared meat foods. In considering the relative advantages of these substances three factors must be borne in mind: (1) the condition of the patient, and the effect upon him which it is desirous to produce; (2) the properties and strength of the solution we wish to give him; and (3) the relative cost of the home-made beef tea and the commercial extract or preparation. The first must be determined by the physician, the second by the chemist and physiologist, and the third by mathematical calculation. We have to determine if the patient requires a stimulating condiment, an easily digestible food, or a combination of the two; if we require to produce an increased katabolism with a temporary manifestation of energy in order to tide over a crisis or a gradual long-continued anabolic process to repair the waste of tissue which has already occurred during an acute illness or which is accompanying a slow wasting disease. It is obvious, for example, that a patient approaching or recovering from the crisis in pneumonia must be treated differently from one who has enteric fever or requires to be fed up to counteract phthisical wasting. Each case must be judged on its merits, and it is not sufficient to order beef tea or meat extract as a part of the rule of thumb "slop" diet.

We shall, therefore, refer to the analyses which were made by Dr. Rideal for THE HOSPITAL and republished in the book "Hospital Expenditure: the Commissariat,"* in order to show the composition of carefully prepared beef tea and then discuss its action, its fate and its influence on the tissues of the patient so that we can determine what clinical conditions call for its administration. It is scarcely necessary to state that beef tea is not a food, for that is a fact universally admitted, but its physiological action is by no means so well known.

Food stuffs can be roughly divided into proteids, fats, carbohydrates, and salts. In beef tea the proteids are represented by albumen and its derivatives, but these are present only in small quantities, which vary according to the method employed in brewing; fats are also present, but only in small quantities; carbohydrates for all practical purposes may be considered absent, but the salts of beef are largely taken into solution. The reason for this is that most of the proteids of meat are insoluble in water, and even the small amount which is soluble in cold water is mainly coagulated and rendered insoluble when the solution is heated. The substances which do enter into solution in the process of making beef tea or extracts are spoken of now by chemists as flesh bases, but much remains to be done by experimental physiologists before we can state what small amount (if any) of nutritive value they possess. But undoubtedly the chief nourishing properties of the meat are left in the hard, dry remnant of meat fibre which remains undissolved and is usually thrown away. Sir William Roberts states "if this is beaten to a paste with a spoon or pounded in a mortar, and duly flavoured with salt and other condiments, it constitutes not only a highly nourishing and agreeable but also an exceedingly digestible form of food."†

The substances which enter into solution and are therefore found in beef tea and meat extracts are, as we stated before, called flesh bases, and consist chiefly of creatin, creatinin, xanthin, hypoxanthin, taurin, sarcolactic acid, and occasionally urea and uric acid are also present. The action of these substances is mainly stimulative and may be compared to that of tea, coffee, or alcohol, but the manner in which they act will be more fully discussed hereafter. Here we will only state that since beef tea and meat extracts consist of the same substances, their action may be considered practically identical, and in order to make a solution of meat extract containing an equal quantity of solids to that usually found in beef tea, it is merely necessary to know the percentage of total solids in the extract. It is easy, therefore, to show that, taking a standard strength of beef tea, we are enabled to imitate it by adding a certain quantity of meat extract to water, and also that it is possible to arrive at a simple formula by which this quantity can be at once determined. We shall then be in a position to discover if the relative cost of beef tea is greater or less than that of a solution of equal quantity, and containing the same weight of solids made from one of the commercial extracts. Different samples of beef tea vary to a large extent according to the quality of the meat and the manner in which it is brewed, so we are compelled to adopt an arbitrary standard. But when we come to consider the nourishing meat preparations, the question becomes far more complicated, for then the weight of digestible proteid food-stuff will have to be taken into account as well as that of the extractives.

* Chapters XXI. and XXII.

† Sir William Roberts "Digestion and Diet," p. 181.

